# Potential of *Zanthoxylum leprieurii* as a source of active compounds against drug resistant *Mycobacterium tuberculosis*

**DOI:** 10.1186/s12906-017-1602-x

**Published:** 2017-02-02

**Authors:** Lydia Bunalema, Ghislain Wabo Fotso, Paul Waako, John Tabuti, Samuel O. Yeboah

**Affiliations:** 1 Department of Pharmacology and Therapeutics, College of Health Sciences, Makerere University, P.O Box 7072, Kampala, Uganda; 2 Department of Organic Chemistry, Faculty of Science, University of Yaoundé I, P.O Box 812, Yaoundé, Cameroon; 3 Department of Environmental management, Makerere University, P.O. Box 7062, Kampala, Uganda; 4 Department of Chemistry, Faculty of Science, University of Botswana, Private Bag UB00704, Gaborone, Botswana

**Keywords:** *Zanthoxylum leprieurii*, Tuberculosis, Isolation, Acridone alkaloids, Antimycobacterial activity

## Abstract

**Background:**

Tuberculosis (TB) is still a global health problem mainly due to development of resistance and co-infection with the Human immune Virus (HIV). Treatment of multi and extensively drug resistant TB requires use of second line drugs which are less efficacious, expensive and very toxic. This has necessitated a need to search for new treatment regimens especially from medicinal plants. *Zanthoxylum leprieurii*, a plant species from *Rutaceae* is used locally in the treatment of tuberculosis in Uganda. The aim of the study was to isolate, identify and characterize bio active compounds from *Z. leprieurii* stem bark with antimycobacterial activity.

**Methods:**

Crude extracts, fractions and compounds from air dried stem bark of Z. *leprieurii* were tested against pan sensitive (H37rv), isoniazid resistant (TMC 301) and rifampicin resistant (TMC 331) strains of *M. tuberculosis* using micro plate alamar blue assay. Isolation of active compounds was done by using column chromatography and thin layer chromatography. They were analysed using nuclear magnetic resonance spectroscopy and mass spectroscopy.

**Results:**

The methanol extract had minimum inhibitory concentrations (MIC) of 47.5, 75.3 and 125.0 μg/ml on the pan sensitive strain, rifampicin resistant and isozianid resistant strains of *M. tuberculosis* respectively. The chloroform extract had MIC values of 260 μg/ml agnaist the pan sensitive strain and 156 μg/ml on the rifampicin resistant strain. Of the sixteen fractions from the methanol extract, fraction Za_4_ (MIC = 6.3 μg/mL, 23.0 μg/mL, 11.7 μg/mL) and Za_6_ (MIC = 11.7 μg/mL 31.2 μg/ml, 31.2 μg/ml) were the most active. Three acridone alkaloids; hydroxy-1, 3-dimethoxy-10-methyl-9-acridone (**1**), 1-hydroxy-3-methoxy-10-methyl-9-acridone (**2**) and 3-hydroxy-1, 5, 6-trimethoxy-9-acridone (**3**) were isolated from Za_4_ and Za_6_. The MIC of compound **3** was found to be 5.1 μg/ml, 4.5 μg/ml and 3.9 μg/ml on H37rv, TMC 331 and TMC 301 while that of **1** was found to be 1.5 μg/ml, 8.3 μg/ml and 3.5 μg/ml respectively.

**Conclusion:**

The results of this study suggest that *Z. leprieurii* is active on resistant strains of *M. tuberculosis* and could be a potential source of new leads against resistant tuberculosis. It also verifies the local use of the plant in treatment of tuberculosis.

**Electronic supplementary material:**

The online version of this article (doi:10.1186/s12906-017-1602-x) contains supplementary material, which is available to authorized users.

## Background

*Mycobacterium tuberculosis*, the bacillus that causes tuberculosis (TB), is one of the leading causes of illness and death worldwide, accounting for 9.6 million new cases and 1.5 million deaths per year [1]. Africa carries the most severe TB burden with a prevalence of 281 cases per 100,000 individuals compared to the global average of 133/100, 000 [2]. Uganda, one of the high TB burden countries, reports approximately 166 new cases per 100,000 people [3]. The high TB burden is attributed to Human Immune Virus (HIV) co-infection in addition to development of multi- and extensively drug resistant strains (MDR and XDR) of TB [3]. Worldwide, MDR TB was reported to account for 5% of all new tuberculosis cases [4] of which 9% was caused by XDR-TB [5].

Treatment of resistant strains of MDR TB presents a challenge globally because it involves use of more than three drugs for a long period which results in non adherence and development of even more resistant strains [6]. While new drugs like bedaquiline and delamanid have recently been discovered and approved for use in MDR TB [7], they are expensive and mostly unavailable in poor resource countries. Furthermore, discovery of these new drugs took approximately 40 years since the last new anti TB drug was introduced. This slow pace in drug development in addition to the rapid emergence and spread of resistant TB calls for continuous search for new agents that are effective against resistant *M. tuberculosis*.

Medicinal plants have long been used for treatment of different ailments in local communities [8]. As such, drugs like chloroquine, artemisinin derivatives and morphine have been derived from different plant sources [9]. *Zanthoxyllum* contains about 549 species distributed worldwide; making it the largest genus in *Rutaceae* [10] *Zanthoxyllum leprieurii,* a plant species belonging to the same family, is traditionally used in the treatment of HIV/AIDS, malaria, urinary infections, rheumatic pain and is used as an antiseptic in Africa [11, 12]. In addition, it is used by communities in Uganda for management of TB and cough related infections [13, 14]. Pharmacological assessment of *Z. leprieurii* could potentially lead to the discovery of new anti-TB agents as well as verify its use among local people in Uganda communities. The aims of the study were to; assess the anti mycobacterial activity of *Z. leprieurii*, to isolate compounds from *Z. leprieurii* and to determine the antimycobacterial activity of the isolated compounds against a pan sensitive, rifampicin resistant and isoniazid resistant strains of *M. tuberculosis.*

## Methods

### Preparation of plant extract

Stem bark of *Z. leprieurii* Guill. & Perr (locally called ‘Munyenye’ in luganda) was collected from Mpigi District in Central Uganda (0° 13′ 38.4708″ N 32° 19′ 29.7264″ E). With the help of a botanist (Paul Segawa from Makerere University Herbarium), the plant was identified and voucher specimen indexed as BL 030 was deposited at the Makerere University Herbarium. The powdered plant material (500 g) was weighed and soaked separately in methanol (1000 ml) and chloroform (1000 ml) for four days with occasional shaking. The filtrates were concentrated under reduced pressure using a rotary evaporator to give 13.25 g and 15.48 g of methanol and chloroform crude extracts respectively.

### Mycobacterial testing

#### Strains

Three preserved strains of *M. tuberculosis* were used and they included; a rifampicin resistant strain (TMC 331/ATCC35838), an isoniazid resistant strain (TMC 301/ATCC 35820) and a pan sensitive strain (H_37_Rv) as a positive control. All the strains were obtained from Joint Clinical Research centre (JCRC) Kampala Uganda.

#### Preparation of inoculums

*M. tuberculosis* was grown on Middle brook 7H10 agar for three weeks and then introduced into 7H9 broth (10 ml) supplemented with 0.2% (v/v) glycerol, oleic acid, albumin, dextrose, catalase (OADC). The inoculum was incubated at 37 °C for 24 h. Turbidity equivalent to 0.5 MC Farland standards (1.5*10^8^ CFU) were prepared using a nephlometer.

#### Preparation of drugs and plant extracts

The plant crude extracts, fractions, pure compounds and standard drugs were prepared in either sterile distilled water or DMSO depending on their solubility. The final concentrations tested ranged from 0.024- 6.250 μg/ml, 1.9–500 μg/ml and 24.4–6250 μg/ml for compounds/drugs, fractions and extracts respectively.

#### Procedure

Susceptibility testing: Micro plate alamar blue assay (MABA) was used as described by Lawal et al., (2011) [15]. Sterile distilled water (200 μl) was added to all outer perimeter wells of sterile 96-well plates to minimize evaporation. The remaining wells received 100 μl of 7H9 broth. One hundred micro liters of double concentration drug solutions were added to each of the wells in rows B to G in column 2 and serial dilutions were made through column 10 using a multi channel pipette.

Each of the wells in rows from 2–11 were inoculated with 100 μl of *M. tuberculosis* bringing the final volume to 200 μl per well. The wells in column 11 were drug free containing only the inoculum and broth and these acted as negative control wells. The plates were incubated at 37 °C for 24 h. The tests were prepared in triplicate for each of the strains used. Thirty micro liters of a freshly prepared alamar blue reagent was added to one of the control wells and further incubated at 37 °C for 24 h. Observation of a color change indicated that there was growth. The dye was then added to all the wells and further incubated for 24 h. The minimum inhibitory concentration (MIC) was defined as the lowest drug concentration which prevented a color change from blue to pink (Additional file 1). The bioassays were performed in a level 3 bio safety laboratory.

#### Phytochemical testing

The methanolic extract was subjected to silica gel column chromatography using a gradient system of *n*-hexane-AcOEt and AcOEt-MeOH as eluents. Fractions of 250 ml each were collected and combined on the basis of their thin layer chromatography profile. Sixteen fractions (Za_1_-Za_16_) were obtained and these were again tested on *Mycobacterium* using the above method. The fractions which showed the lowest MIC values (i.e. Za_4_ and Za_6_) were isolated using repetitive preparative TLC to obtain compounds **1**, **2** and **3**. These were analyzed using intensive 1D and 2D nuclear magnetic resonance (NMR) spectroscopy.

### General experimental procedures

Analytical and Preparative TLC were performed respectively using silica gel 60 PF254 + 366 (Merck) and silica gel 60-F254 precoated aluminum sheets (Merck). The plates were visualized under UV (254 and 366 nm) and revealed by spraying with vanillin-sulphuric acid (Additional file 2). NMR spectra were recorded on Bruker DMX Avance 300 MHz instrument equipped with an auto-tune probe and using the automation mode aided by chloroform (CDCl_3_) as solvent and internal standards.

## Results

### Antimycobacterial activity of fractions and crude extracts

The MIC values of the methanol crude extract were 47.5 μg/ml, 75.3 μg/ml and 125.0 μg/ml against the pan sensitive, rifampicin resistant and isoniazid resistant strains respectively (Table 1). The chloroform crude extract had MICs of 260.0 μg/ml and 156.3 μg/ml on the pan sensitive and isoniazid resistant strains respectively. The methanol extract showed a relatively lower MIC value as compared to the chloroform extract.

**Table 1:** Mean MIC values of extracts and fractions from *Z. leprieurii* on different species of *M. tuberculosis*

		MIC (μg/ml)	
	Pan sensitive strain H37R_V_	Rifampicin resistant strain TMC 331	Isoniazid resistant strain TMC 301
Crude extracts			
Chloroform extract	260.0	>6250	156.3
Methanol extract	47.5	75.3	125.0
Fractions			
Za_1_	31.3	31.3	78.2
Za_2_	94.5	31.3	63.2
Za_3_	>500	16.4	>500
Za_4_	6.3	23.0	11.7
Za_5_	31.3	31.3	94.1
Za_6_	11.7	31.3	31.3
Za_7_	94.1	94.1	>500
Za_8_	63.2	31.3	>500
Za_9_	63.2	>500	>500
^a^Za _10_	>500	>500	>500
Rifampicin	2.0	-	2.0
Isoniazid	4.0	4.0	-

^a^Za _10_ = Za_11−_Za_16_ with MIC >500 ug/ml

Sixteen fractions from the methanol crude extract were further tested. The fractions that had MICs ≤ 100 μg/ml on all the tested strains were; Za_1_, Za_2_, Za_3_, Za_4_ and Za_6_ (Table 1). Fraction Za_4_ had MICs of 6 μg/ml, 23 μg/ml and 11.7 μg/ml on the pan sensitive, rifampicin resistant and isoniazid resistant strains respectively. Za_6_ had MICs of 11.7 μg/ml on the pan sensitive strain well as on resistant strains its MIC was31.2 μg/ml. The MIC of fractions Za_10-16_ was beyond the tested concentration of 500 μg/ml.

#### Spectral data of isolated compounds

**Fig. 1:**
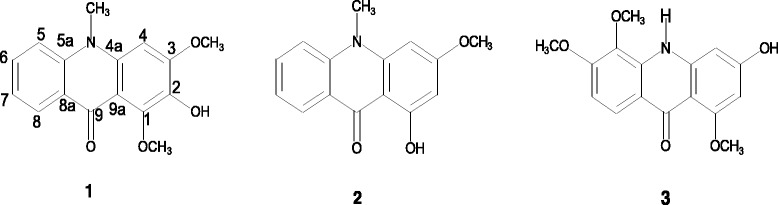
Chemical structure of compounds 1, 2 and 3

Three compounds, ***1***-***3*** *(*Fig. 1) were isolated from fractions Za_4_ and Za_6_ of the methanol extract. Their spectral data is displayed below (Additional file 3):

- Compound **1**: yellow needle like crystals (8.2 mg); ^1^H NMR (300 MHz, CDCl_3_), δ_H_ (m, *J* in Hertz): 6.32 (s, H-4), 7.55 (brd, *J* = 9 Hz, H-5), 7.77(ddd, *J* = 8.7, 6.9, 1.8 Hz, H-6), 7.36 (m, H-7), 8.50(dd, *J* = 7.8, 1.5 Hz, H-8), 3.89(s, N-CH_3_), 3.98 (s, 1-OCH_3_), 4.07 (s, 3-OCH_3_); ^13^C NMR (75 MHz, CDCl_3_): 156.2 (C-1), 130.2 (C-2), 159.4 (C-3), 86.7 (C-4), 140.5 (C-4a), 114.6(C-5), 142.1(C-5a), 134.0 (C-6), 121.5(C-7), 126.7(C-8), 120.8 (C-8a), 180.7 (C-9), 105.9 (C-9a), 34.1(N-CH_3_), 60.8 (1-OCH_3_), 56.0 (3-OCH_3_); ESI-MS ([M] ^+^ at *m/z* 285.4) calcd for C_16_H_15_NO_4_
- Compound **2**: Yellow powder (7.2 mg); ^1^H NMR (300 MHz, CDCl_3_), δ_H_ (m, *J* in Hertz): 6.32(d, *J* = 2.1 Hz, H-2), 6.54(d, *J* = 2.2 Hz, H-4), 7.25 (m, H-5), 7.25 (m, H-6), 7.35 (m, H-7), 8.39 (dd, *J* = 8.1, 1.6 Hz, H-8), 3.93 (s, N-CH_3_), 3.97 (s, 3-OCH_3_); ^13^C NMR (75 MHz, CDCl_3_): 165.3 (C-1), 89.6 (C-2), 166.3 (C-3), 89.5 (C-4), 142.5 (C-4a), 115.4 (C-5), 145.0 (C-5a), 134.1 (C-6), 121.2 (C-7), 126.9 (C-8), 120.7 (C-8a), 180.4 (C-9), 104.8 (C-9a), 33.8 (N-CH_3_), 55.2 (3-OCH_3_); ESI-MS([M]^+^ at *m/z* 255.3) calcd for C_15_H_13_NO_3_
- Compound **3**: Orange powder (5.0 mg); ^1^H NMR (300 MHz, CDCl_3_), δ_H_ (m, *J* in Hertz): 8.07(d, *J* = 9.6 Hz, H-8), 7.64 (d, *J* = 2.7 Hz, H-4) 7.29 (d, *J* = 9.6 Hz, H-7) 7.10 (d, *J* = 2.7Hz, H-2), 4.49 (s, 5-OCH_3_), 4.17 (s, 1-OCH3), 4.08 (s, N-H)**;** ^13^C NMR (75 MHz, CDCl_3_): 157.3 (C-1), 102.1 (C-2), 164.3 (C-3), 104.7 (C-4), 143.1 (C-4a), 141.4 (C-5), 142.0 (C-5a), 152.2 (C-6), 112.2 (C-7), 118.2 (C-8), 115.0 (C-8a), 185.1 (C-9), 104.7 (C-9a), 59.1 (1- OCH_3_), 61.7 (5-OCH_3_), 56.9 (6-OCH_3_)

#### Antimycobacterial activity of compounds

Compound **1**, **2** and **3** were screened for activity against *M. tuberculosis.* Compound **1** had MICs of 1.5 μg/ml, 3.5 μg/ml and 8.3 μg/ml on the pan sensitive, isoniazid resistant and rifampicin resistant strains respectively (Table 2). Compound **3** had MICs of 5.1 μg/ml, 4.5 μg/ml and 3.9 μg/ml on the pan sensitive, isoniazid resistant and rifampicin resistant strains of *M. tuberculosis* respectively while the MIC of compound **2** was beyond the tested concentration, 6.250 μg/ml.

**Table 2:** Mean MIC Values of isolated compounds 1, 2 and 3 on three strains of M. tuberculosis

	Antimycobacterial activity MIC (μg/ml)		
Compound	Pan sensitive strain (H37R_V_)	Rifampicin resistant strain (TMC 331)	Isoniazid resistant strain (TMC 301)
1	1.5 ± 0.8	5.9 ± 3.2	3.2 ± 1.7
2	>6.250	>6.250	>6.250
3	5.1 ± 1.1	4.1 ± 0.0	3.9 ± 0.6
Isoniazid	2.0 ± 0.0	4.0 ± 2.1	-
Rifampicin	4.0 ± 0.0	-	2.0 ± 0.0

± Standard deviation

## Discussion

>The search for new anti TB drugs is continuing to be the focus for most research globally due to development of multi drug and extensively drug resistant strains of *M. tuberculosis*. Although drugs like bedaquiline and delamanid have recently been discovered and are used in treatment of MDR TB [7], they are equally expensive and unavailable in poor resource countries. Furthermore reports of totally drug resistant TB in countries like India and South Africa are threatening to make the disease untreatable [16, 17]. In this study, the anti mycobacterial activity of the crude extracts, fractions and compounds from *Z. leprieurii* were evaluated to help demonstrate the plant’s potential as an alternative source for new anti TB agents. The extracts/fractions were considered to be active if the MICs were ≤ 100 μg/ml [18, 19] while the pure compounds were considered to have antimycobacterial activity if their MIC ≤ 6.25 μg/ml; the maximum tested concentration. In the same way an extract, fraction or compound was considered not to be active if the MIC value was beyond the tested concentration.

Both crude extracts from *Z. leprieurii* showed some inhibitory activity with the methanol extract being more active against all the *M. tuberculosis* strains as compared to the chloroform extract. This could be due to numerous compounds extracted by methanol which is believed to be an efficient solvent during extraction [20–22]. The methanol extract was fractionated and fractions Za_4_ and Za_6_ were the most active against all the tested strains; implying that they could possibly contain compounds which could be active on MDR TB warranting further phytopharmacological analysis. However, the activity of the crude extracts was generally lower than that of the pure drugs; isoniazid and rifampicin. The low anti-TB inhibition exhibited by the crude extracts could be due to presence of impurities which may interfere with and reduce the potency of the extracts [23]. Over all, purification of *Z. leprieurii* methanol extract yielded fractions that showed substantial activity than the crude extracts especially against the pan sensitive strain. Among these, fraction Za_4_ (MIC = 6.3 μg/ml) was seven times more potent than the methanol crude extract (MIC = 47.5 μg/ml).

Three known acridone alkaloids that is; 2-hydroxy-1, 3-dimethoxy-10-methyl-9-acridone (**1**), 1-hydroxy-3-methoxy-10-methyl-9-acridone (**2**) and 3-hydroxy-1, 5, 6-trimethoxy-9-acridone (**3**) were isolated from fractions Za_4_ and Za_7_. Their structures were confirmed by analyzing their spectral data and also through comparison with already published work [24–26]. When screened for anti mycobacterial activity, compound **1** was found to have a lower MIC value (MIC = 1.5 μg/ml) than both isoniazid (MIC = 0.2 μg/ml) and rifampin (MIC = 0.4 μg/ml); two first line drugs that form a backbone in TB treatment [27]. This shows that there is potential for further development of compound **1** into new anti TB agents that could be an alternative to the two most important first line drugs in TB treatment. On the contrary, Compound **2** was inactive on *M. tuberculosis*. The variation in activity could not be verified in this study however it is thought that it could be linked to the number and positions of hydroxyl and methoxy groups on rings A and B. Previous studies have shown that the antimycobacterial activity of alkaloids is affected by the number and positions of functional groups [28].

Previous studies done on Cameroonian spices revealed that *Z. xanthoxyloides* and *Z. macrophylla* showed weak activity with MICs of 1.024 mg/ml against H37Rv and H37Ra [29]. However a study by Luo et al., [30] showed that extracts from *Z. capense* had antimycobacterial activity. In addition, decarine, a benzophenanthridine alkaloid was isolated and found to inhibit growth of *M. tuberculosis* within macrophages with MIC of 1.6 μg/ml on the pan sensitive strain. Compound **3** has been shown to have cytotoxic effects (IC50 of 86 μM) against liver cancer cell lines by inhibiting glycosyltransferase and aromatase enzymes in the liver [31]. In addition compound **2** had a moderate activity of 33% mortality against *Anopheles gambiae* larvae at a concentration of 1000 ppm [31]. In a review by Kishore et al., (2009) [32], different types of alkaloids have been shown to have anti mycobacterial activity however no study has reported activity of acridone alkaloids on resistant strains of TB. Our results add to other plant derived compounds from genus Zanthoxyllum which could be further explored as anti TB agents.

## Conclusion

Our results show that Pharmacological assessment of *Z. leprieurii* could potentially lead to the discovery of new anti TB agents. Bioassay guided isolation of *Z. leprieurii* afforded three acridone alkaloids; two of which showed activity on *M. tuberculosis* resistant strains. These findings also provide some scientific evidence to support, to some extent, the ethnomedicinal use of *Z. leprieurii* as traditional antitubercular remedies in Uganda communities though more studies on the in-vivo antimycobacterial activity and toxicity of the bio active compounds is needed.

## Additional files

Additional file 1: Figure showing one of the results as they were observed on microplate. (DOCX 421 kb)
Additional file 2: Figure showing purity of the isolated compounds as separated by TLC plates. (DOCX 124 kb)
Additional file 3: Proton NMR spectra of compounds 1, 2 and 3. (DOCX 208 kb)

## Abbreviations

1D: One dimensional
2D: Two dimensional
AIDS: Acquired immune deficiency syndrome
HIV: Human immune virus
JCRC: Joint clinical research centre
MABA: Micro plate alamar blue assay
MDR: Multi drug resistance
MIC: Minimum inhibitory concentration
NMR: Nuclear magnetic resonance
TB: Tuberculosis
TLC: Thin layer chromatography
WHO: World Health Organisation
XDR: Extensive drug resistance
