# Supplementary figures and images for: Potential of Zanthoxylum leprieurii as a source of active compounds against drug resistant Mycobacterium tuberculosis
Source: BMC Complement Altern Med. 2017 Feb 2;17:89. doi: 10.1186/s12906-017-1602-x (PMC5289037; doi:10.1186/s12906-017-1602-x)

Figure showing one of the results as they were observed on microplate


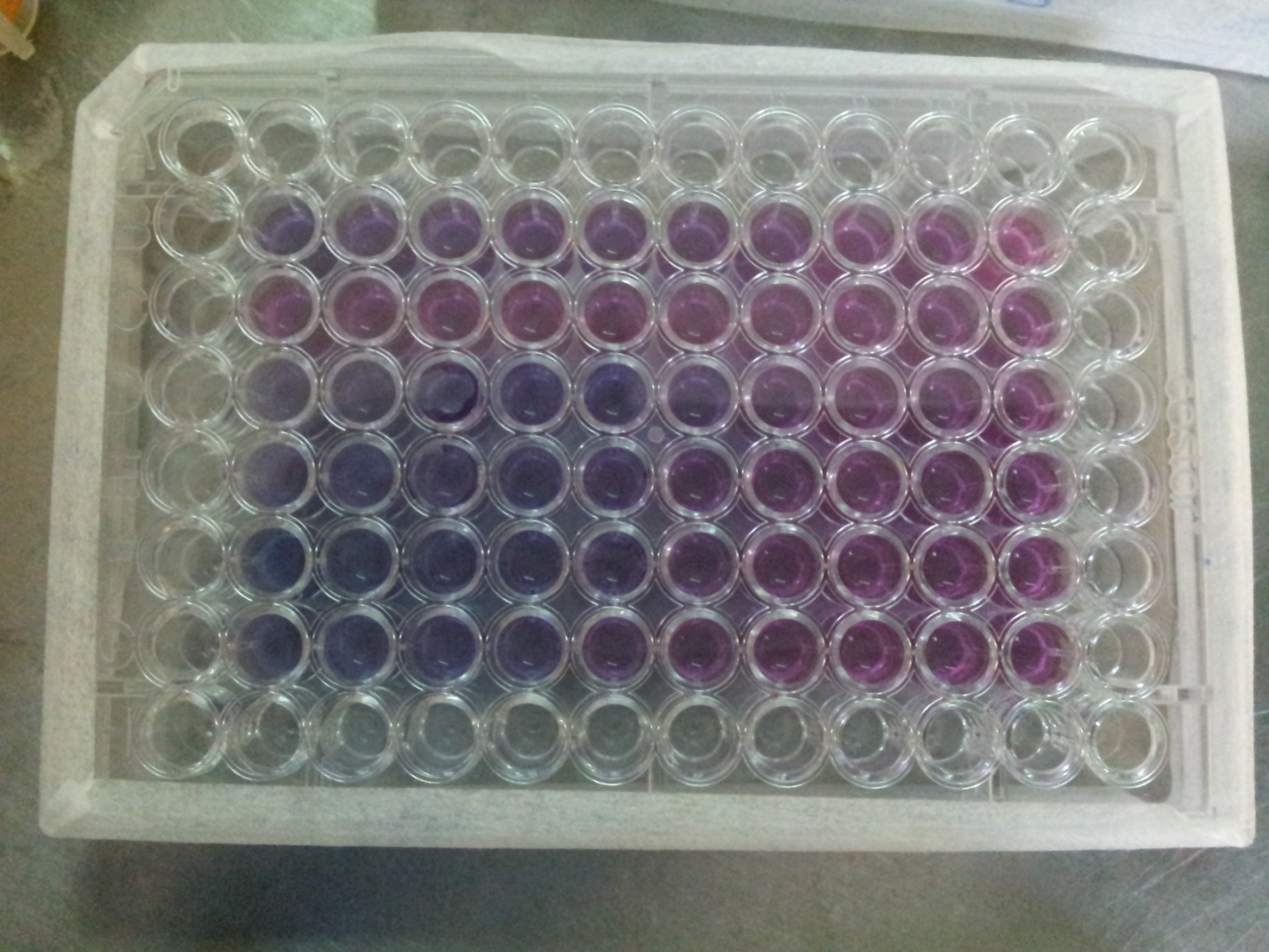

Supplement: Additional file 1: — Figure showing one of the results as they were observed on microplate. (DOCX 421 kb) [file 12906_2017_1602_MOESM1_ESM.docx]

Figure showing purity of the isolated compounds as separated by TLC plates


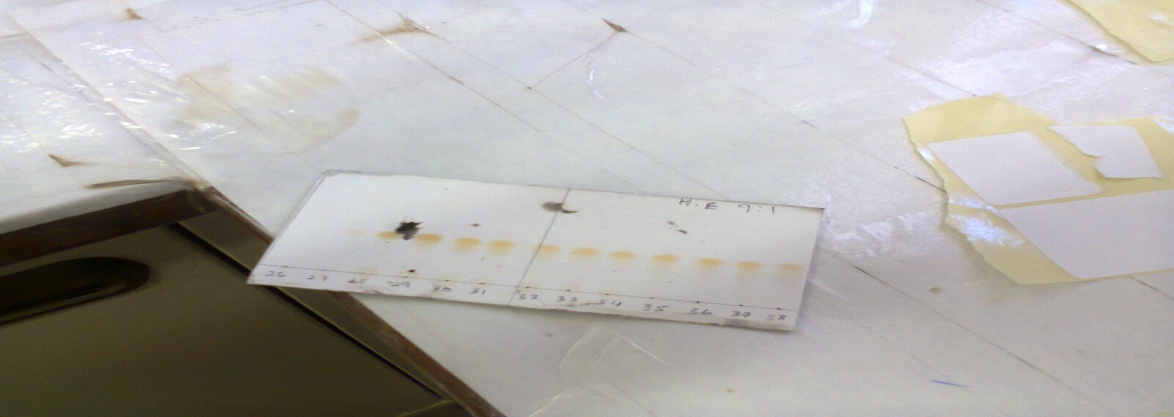

Supplement: Additional file 2: — Figure showing purity of the isolated compounds as separated by TLC plates. (DOCX 124 kb) [file 12906_2017_1602_MOESM2_ESM.docx]
