# Supplementary material for: Potential of Zanthoxylum leprieurii as a source of active compounds against drug resistant Mycobacterium tuberculosis
Source: BMC Complement Altern Med. 2017 Feb 2;17:89. doi: 10.1186/s12906-017-1602-x (PMC5289037; doi:10.1186/s12906-017-1602-x)
Supplement: Additional file 3: — Proton NMR spectra of compounds 1, 2 and 3. (DOCX 208 kb) [file 12906_2017_1602_MOESM3_ESM.docx]

Proton NMR spectra of compounds 1, 2 and 3

Proton Nmr spectra of compound **3**

Proton NMR Spectra of compound **2**


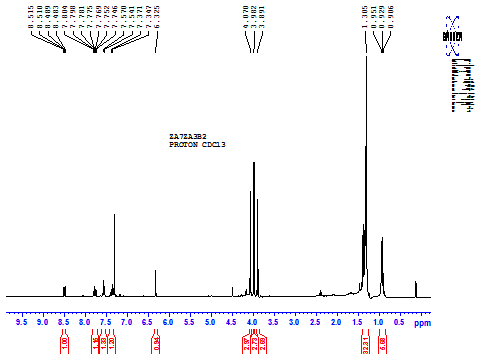


Proton Nmr Spectra of compound **1**
